# The Circadian Clock in Glioblastoma: Mechanisms and Therapeutic Potential

**DOI:** 10.3390/clockssleep8040059

**Published:** 2026-09-24

**Authors:** Samaneh Ghorbani Shirkouhi, Farzin Kamari, Poul Flemming Høilund-Carlsen, Morten Blaabjerg, Abass Alavi, Frantz Rom Poulsen, Mona-Elisabeth R. Revheim, Anderson Matheus Pereira da Silva, Sasan Andalib

**Affiliations:** 1 Independent Researcher, Rasht 4173143385, Iran; samaneh.shirkouhi@gmail.com; 2 Department of Neurophysiology, Institute of Physiology, Eberhard Karls University of Tübingen, 72074 Tübingen, Germany; farzin.kamari@uni-tuebingen.de; 3 Department of Nuclear Medicine, Odense University Hospital, 5000 Odense, Denmark; pfhc@rsyd.dk; 4 Research Unit of Clinical Physiology and Nuclear Medicine, Department of Clinical Research, Faculty of Health Sciences, University of Southern Denmark, 5230 Odense, Denmark; 5 Research Unit of Neurology, Department of Clinical Research, Faculty of Health Sciences, University of Southern Denmark, 5230 Odense, Denmark; morten.blaabjerg1@rsyd.dk; 6 Department of Neurology, Odense University Hospital, 5000 Odense, Denmark; 7 Department of Radiology, Perelman School of Medicine, University of Pennsylvania, Philadelphia, PA 19104, USA; abass.alavi@pennmedicine.upenn.edu; 8 Department of Neurosurgery, Odense University Hospital, 5000 Odense, Denmark; frantz.r.poulsen@rsyd.dk; 9 Brain Research Interdisciplinary Guided Excellence (BRIDGE), University of Southern Denmark, 5230 Odense, Denmark; 10 The Intervention Centre, Oslo University Hospital, 0372 Oslo, Norway; monar@ous-hf.no; 11 Institute of Clinical Medicine, Faculty of Medicine, University of Oslo, 0372 Oslo, Norway; 12 Department of Physiology and Pharmacology, Federal University of Pernambuco, Recife 50670-420, PE, Brazil; anderson.matheuss@ufpe.br

**Keywords:** circadian rhythm, glioblastoma, molecular mechanisms, therapeutic approaches

## Abstract

Glioblastoma (GBM) is an aggressive brain cancer in adults. Circadian rhythms regulate the sleep–wake cycle, which influences the body’s physiological functions. Disruption of circadian rhythms is associated with certain neurological diseases, including GBM. The present narrative review highlights the relationship between circadian rhythm and GBM, the related mechanisms, and the therapeutic approaches against GBM. It also discusses how alterations in the expression of core circadian genes contribute to glioma genesis, especially GBM, and how restoring normal circadian gene function may suppress GBM progression. Additionally, chronotherapy could be considered a potential therapeutic approach for GBM. Aligning chemotherapy and radiotherapy with the internal clock of each patient may have the potential to increase the efficacy of treatments. A better understanding of the potential mechanisms underlying the association between circadian rhythm and GBM pathogenesis may help guide future circadian-based therapeutic approaches; however, further investigation is required.

## 1. Introduction

Circadian rhythm in mammals is an approximately 24 h internal clock in the suprachiasmatic nucleus (SCN) of the brain, which regulates the sleep–wake cycle. The sleep–wake cycle influences various physiological functions, including eating habits, body temperature, and hormone secretion [1]. Circadian rhythm disturbances (CRDs) are associated with metabolic, psychiatric, and cardiovascular disorders [1]. CRDs have also been linked to cancer [2]. The association between disturbance in the rhythmic expression of core-clock genes and glioblastoma (GBM) has been suggested [3].

GBM is the most common malignant primary central nervous system (CNS) tumor in adults, with an incidence of approximately 3.19 cases per 100,000 population [4]. GBM expansion occurs through various genetic and molecular aberrations, which lead to significant alterations in signaling pathways [5].

According to the 2021 World Health Organization (WHO) classification of tumors of the CNS, GBM is defined as an isocitrate dehydrogenase (IDH)-wildtype diffuse astrocytic glioma, WHO grade 4, lacking mutations in *IDH1* and *IDH2* genes [6]. It is histologically characterized by microvascular proliferation or necrosis and is molecularly defined by the presence of telomerase reverse transcriptase (*TERT)* promoter mutation, epidermal growth factor receptor (*EGFR)* gene amplification, or the combined chromosome 7 gain/chromosome 10 loss (+7/−10 copy number changes) [6]. It has been suggested that neural stem cells (NSCs) may give rise to GBM [7]. Additionally, GBM may arise from more differentiated glial populations, such as astrocytes or oligodendrocyte precursor cells (OPCs) [7,8].

Patients with malignant gliomas experience symptoms, including headache, seizures, memory loss, focal neurologic deficits, confusion, and personality changes [9]. GBM symptoms are more severe than those of other gliomas.

The standard treatment for glioblastoma relies on combining surgical tumor resection, radiotherapy, and chemotherapy in various combinations; yet, technological advances, especially in biotechnology, are enabling the development of newer and more precise therapies [10].

It is important to distinguish sleep disturbances (SDs) in patients with GBM [11] from tumor-related CRD. SD may be a result of tumors or the effect of medications [12]. In contrast, tumor-related CRD may be associated with altered expression of circadian genes and disruption in circadian signaling within GBM cells that can impact proliferation [13], stemness [14], angiogenesis [15], and deoxyribonucleic acid (DNA) repair [16]. Although these processes may influence each other, they should not be considered equivalent.

GBM remains one of the most lethal human malignancies. Its highly aggressive behavior and resistance to chemotherapy and radiotherapy have limited the effectiveness of current therapeutic approaches. There are no curative treatments for GBM, and despite numerous therapeutic advances, the survival of patients with GBM remains poor [17]. Compared with many other primary brain tumors, GBM represents a more urgent therapeutic need because of its highly aggressive nature and persistently poor prognosis [18]. Circadian-based interventions may be a promising compensatory approach to improve treatment efficacy. The present narrative review discusses the relationship between circadian rhythm and GBM and highlights the underlying mechanisms and the therapeutic potential for GBM.

## 2. Literature Search Strategy

In this narrative review, PubMed and Google Scholar were used through keywords such as glioblastoma, brain cancer, circadian rhythm, clock genes, chronotherapy, time of day, radiotherapy, and temozolomide (TMZ) to identify literature. No time limitation was applied during the literature search. Afterward, publications were selected based on their relevance and contribution to the overall narrative of this review.

## 3. Molecular Architecture of the Circadian Clock

The pacemaker of the circadian rhythm is the SCN of the hypothalamus. The circadian system comprises the central and peripheral clocks. While the SCN serves as the central circadian pacemaker, functional molecular clock machinery is also widely expressed in most peripheral organs and tissues [19], generating local circadian rhythms and regulating tissue-specific physiological processes [20]. The central clock in the SCN can work independently, but the peripheral clock in the tissues is synchronized through various signaling molecules [2].

The molecular basis of circadian rhythms in mammals is the transcription–translation feedback loop (TTFL). TTFL consists of two activators, circadian locomotor output cycles kaput (*CLOCK*) and basic helix-loop-helix ARNT-like 1 (*BMAL1*), and two inhibitors, cryptochromes (*CRYs*) and period circadian regulators (*PERs*) [2,21,22]. The circadian proteins CLOCK and BMAL1 heterodimerize in the cytoplasm and are then transferred to the nucleus. CLOCK/BMAL1 complex interacts with enhancer box (E-box) elements in the promoters of target genes, including *CRY* and *PER*. PER and CRY proteins form a complex in the cytoplasm. This complex is transferred to the nucleus and inhibits the transcriptional activity of the CLOCK/BMAL1 complex [23]. Additionally, the CLOCK/BMAL1 complex activates the transcription of nuclear receptor subfamily 1 group D members (NR1D or REV-ERBα/β) and retinoic acid receptor-related orphan receptors (RORα/β/γ). On the other hand, REV-ERBs inhibit transcription of *BMAL1* by blocking the ROR element (RORE) binding site of the *BMAL1* promoter. Conversely, RORs promote the transcription of *BMAL1* [24,25,26].

## 4. The Role of the Circadian Clock in Cell Cycle and DNA Damage

The circadian clock participates in the cell cycle, DNA repair, and DNA damage response [27]. The circadian clock affects the DNA damage checkpoint response by controlling the transcription of the checkpoint proteins [28]. In addition, the core clock system regulates genes involved in generating, sensing, and transducing the checkpoint signals and cell-cycle arrest [27]. *PER1* has been implicated in cell-cycle control [29]. Overexpression of *PER1* led to sensitization of human cancer cells to programmed cell death (apoptosis) induced by DNA damage, whereas inhibition of *PER1* reduced apoptosis in similarly treated cells [29]. Even samples of patients with cancer showed a general reduction in *PER1* levels [29]. *PER2* binds directly to p53, a crucial tumor suppressor protein, and controls p53-mediated transcriptional activity [30]. PER2 binds to p53 and forms a stable complex with murine double minute 2 (Mdm2), a negative regulator of p53, thereby preventing Mdm2-mediated p53 degradation and promoting p53 expression [30]. Transcription of the *Mdm2* gene is activated by p53 [31,32]. Stimulation of p53 degradation by MDM2 protein inhibits MDM2 production [31,32]. *Per2* expression was shown to be regulated by binding of p53 to its response element in the *Per2* promoter [33]. The p53 response element in the *Per2* promoter overlaps with the E-box element for BMAL1/CLOCK binding [33]. It was shown that p53 suppresses *Per2* expression by blocking BMAL1/CLOCK binding to the *Per2* promoter [33].

Sun et al. [34] indicated that the overexpression of *PER2* in human K562 leukemia cells results in cell-cycle arrest at the G2/M phase. They also observed an increase in apoptosis, confirmed by characteristic morphological changes and DNA fragmentation. Similarly, transfection of *Per2* in mice with leukemia suppressed cellular proliferation and accelerated apoptosis of K562 cells [34].

Astone et al. [35] found that impairing the endothelial cell-specific function of *Bmal1* leads to angiogenic defects in neonatal mouse vascular tissues and in adult tumor angiogenic settings. Downregulation of *Bmal1* or *Clock* in hepatocellular carcinoma cells xenografted into mice induced apoptosis and cell-cycle arrest at the G2/M phase [36]. Also, overexpression of *Bmal1* suppressed the proliferation of nasopharyngeal carcinoma cells, whereas its knockdown had the reverse effects [37]. Interestingly, overexpression of *Bmal1* suppresses tumor growth in xenografted mice, whereas *Bmal1* knockdown promotes tumor growth [37]. *Bmal1* overexpression also promotes expression of p53 and p21, while its knockdown suppresses p53 and p21 expression [37].

A study investigated the relationship between cellular response to DNA damage and endogenous PER2 protein expression levels in human normal fibroblasts (CCD-34Lu) [38]. CCD-34Lu received serum shock to induce circadian oscillations of the PER2 protein. The cells were then irradiated with γ-rays (dose rate of 2.8 Gy/min) at times related to the trough and peak expression of the PER2 protein. The results showed effects on clonogenic cell survival, double-strand break (DSB) repair kinetics, and TP53 protein levels in cells irradiated at the trough rather than in those irradiated at peak expression of the PER2 protein [38].

Overall, it may be implied that circadian clock components influence different aspects of biological processes, including cell-cycle progression, apoptosis, DNA repair, and angiogenesis. Variation in the role of individual clock genes suggests complementary functions of these genes and highlights the complexity of their regulation in different tissues.

Figure 1 summarizes the association between circadian genes and cancer at the molecular level.

## 5. Circadian Dysregulation in Cancer

In mammals, the circadian clock regulates numerous biological processes. There is evidence that circadian clock genes act as tumor suppressors, which play a role in retarding cancer development. Circadian clocks are thought to influence the pathways involved in cancer development, including cell-cycle control, apoptosis, and DNA-damage response [39].

Single-nucleotide polymorphisms (SNPs) in *CLOCK*, *BMAL1*, *PER1*, *PER2*, *PER3*, *CRY1*, and *CRY2* genes are proposed to be associated with increased susceptibility to prostate [40], breast, ovarian [41], and pancreatic cancers [42].

## 6. Circadian Rhythm Disturbances in Brain Cancers

There is clinical and experimental evidence regarding CRD in brain cancers. A cross-sectional study explored the prevalence of SD in a population of patients with primary brain tumors [43]. In this study, 424 patients with a mean age of 49 years and 58% with high-grade gliomas (HGGs) were included. In 19% of the patients, moderate-to-severe SD was reported, which was associated with younger age, poor Karnofsky performance status, and tumor progression on magnetic resonance imaging (MRI) [43]. Miaskowski et al. [44] evaluated the association between sleep parameters and fatigue in patients with various cancers, including brain cancer, at the initiation of radiation therapy. Nocturnal sleep/rest, daytime wake/activity, and circadian activity rhythm parameters were measured using actigraphy, the Pittsburgh Sleep Quality Index, the General Sleep Disturbance Scale, and the Lee Fatigue Scale. Between 30% and 50% of patients experienced SD in sleep–wake circadian activity rhythms at the initiation of radiation therapy [44].

It has been suggested that CRD and SD are common in patients with brain tumors and may affect their neurological function and quality of life.

## 7. Circadian Genes in Glioblastoma

The evidence shows an association between chronic CRD and the risk of glioma [45]. Trebucq et al. [46] investigated the role of *Bmal1* in a nude mouse model of human GBM. The tumors were generated from LN229 cells with knocked-down *Bmal1*, which were orthotopically xenografted into the dorsal striatum. Disruption of *Bmal1* in the tumor resulted in reduced survival, increased tumor growth and CD44 expression, worsened motor performance, and increased glial fibrillary acidic protein (GFAP) expression in astrocytes within the tumor microenvironment [46].

Dong et al. [47] reported that downregulation of *BMAL1* or *CLOCK* in glioma stem cells (GSCs) induces cell-cycle arrest and apoptosis. Also, downregulation of *Bmal1* or *Clock* in GSCs before implantation into the frontal lobes of immunocompromised mice led to an approximately twofold increase in the lifespan of tumor-bearing mice [47]. In addition, SR9011 and SR9009, which are small-molecule agonists of nuclear receptors REV-ERBα/β, and KL001, a stabilizer of CRY, were shown to downregulate the stem cell factors and reduce GSC growth. A combination of SR9011 and KL001 further induced synergistic anti-tumor efficacy. Also, KL001 reduced the expression of *PER1* and *PER2* and elevated BMAL1 protein levels in GSCs [47]. Additionally, a mouse model of GBM was used to determine the efficacy of SHP656, a potential CRY stabilizer [47]. SHP656 was well-tolerated, with no weight loss or behavioral changes in mice [47].

Ma et al. [14] suggested that *PER2* mRNA expression and PER2 protein levels are downregulated in GSCs. Following in vitro and in vivo overexpression of *PER2*, they also reported a GSC arrest at the G_0_/G_1_ phase, suppressing cell proliferation, stemness potential, and invasion ability via the Wnt/β-catenin signaling pathway. Hence, they proposed that *PER2* plays a role in the malignant process of glioma and may serve as a target in therapeutic approaches [14].

A previous study revealed that the CLOCK-BMAL1 complex may regulate the interaction between GSCs and endothelial cells in the GBM mouse model and in tumor samples from patients with GBM [48]. In this study, periostin (POSTN) was suggested as a pro-angiogenic factor that promotes tumor angiogenesis in endothelial cells through the activation of TANK-binding kinase 1 (TBK1). CLOCK-olfactomedin-like 3 (OLFML3)-hypoxia-inducible factor 1-alpha (HIF1α) axis was shown to regulate this process [48].

Another study indicated that circadian clock genes are dysregulated in glioma and are associated with glioma prognosis and tumor progression [49]. Silencing the timeless circadian regulator *(TIMELESS*) disrupted circadian clock gene expression and induced cell-cycle arrest at the G0/G1 phase [49]. These findings suggest that disruption in circadian signaling may contribute to glioma progression [49].

To assess the anti-glioma role of retinoic acid receptor-related orphan receptor A (RORA), in a study, Jiang et al. [13] utilized several methods: a cell viability assay, an EdU assay, a neurosphere formation assay, and xenograft experiments. The results showed that *RORA* was expressed at low levels in glioma. Also, an overexpression of *RORA* inhibited the proliferation and tumorigenesis of glioma cell lines and GSCs. It seems that this process occurs through the inhibition of the tumor necrosis factor alpha (TNFα)-mediated nuclear factor kappa B (NF-κB) signaling pathway [13]. Kaplan–Meier survival was used to measure the median survival time of clinical samples from glioma patients. The survival time of patients with high *RORA* expression was greater than that of patients with low *RORA* expression. Micro ribonucleic acid (RNA)-18a (miR-18a) promoted glioma growth through the degradation of the *RORA* gene [13]. The authors suggested that the overexpression of miR-18a may be the cause of low expression of *RORA* in glioma [13].

Circadian clocks exert complementary functions rather than identical functions in GBM biology. On the other hand, variation in approaches used to manipulate circadian genes may explain variability in the reported underlying signaling pathways.

## 8. Chronotherapy in Glioblastoma

Chronotherapy refers to the optimum time of drug administration based on the body’s circadian rhythms. Chronotherapy is performed in two different ways: first, alteration in the sleep–wake cycle of patients aiming to improve pathologies; and second, considering the circadian rhythms of a patient to enhance therapeutics [50].

Slat et al. [51] examined the variation in the efficacy of TMZ on human and mouse GBM cells based on the time of its administration. They found that circadian treatment time affects the sensitivity of murine GBM tumor cells to TMZ in vitro. In addition, it was observed that near the daily peak of *Bmal1* expression, the most TMZ-induced DNA damage response, activation of apoptosis, and growth inhibition occur [51]. The authors indicated that tailoring TMZ administration to the peak of *Bmal1* expression in tumor cells increases TMZ efficacy. Moreover, *Bmal1* deletion led to the elimination of the circadian rhythm in gene expression, activation of apoptosis, and growth inhibition induced by TMZ [51]. In another study, it was hypothesized that the efficacy of TMZ may depend on the time of day, DNA repair enzymes, and O^6^-methylguanine-DNA methyltransferase (MGMT), a critical DNA repair protein, activity in murine and human models of GBM [52]. In vitro recordings showed an intrinsic circadian rhythm in the expression of *Bmal1*, *Per2*, and *Mgmt* in GBM cells. These GBM cells were more susceptible to TMZ at the daily peak of *Bmal1* transcription [52]. Gonzalez-Aponte et al. [53] assessed daily rhythms in *MGMT* promoter methylation in GBM in vitro and *MGMT* methylation in human GBM biopsies, which were collected at different times of day. Additionally, MGMT and BMAL1 protein levels were measured in GBM cells collected at four-hour intervals. MGMT protein levels were highest at the time of maximal TMZ efficacy in vitro [53]. Using GBM cell lines and pharmacological manipulation of *BMAL1*, *NR1D1* (*REV-ERBα*), and *PER2*, Nelson et al. [54] indicated that TMZ sensitivity depends on the time of day. Disruption of clock genes decreased TMZ efficacy [54]. The study mentioned that there is a strong relationship between circadian phenotypes and TMZ pharmacodynamics in GBM [54].

Taken together, these preclinical studies indicate that the efficacy of TMZ not only depends on drug administration but is also influenced by the molecular circadian state of GBM cells. Oscillations in core circadian clock components seem to regulate DNA damage response, apoptosis, and MGMT-mediated repair pathways. These findings may suggest chronotherapy in TMZ administration as a biological determinant in the efficacy of chemotherapy.

In addition, in vivo administration of TMZ in the morning reduced tumor size and increased body weight compared to its administration in the evening. Damato et al. [55], in their retrospective study, reviewed the records of patients with newly diagnosed GBM who had surgery and chemoradiation and received TMZ in the morning or evening. One of the characteristics studied between the morning and evening groups was baseline steroid use, which did not differ significantly. The patients receiving TMZ in the morning experienced longer overall survival than those receiving it in the evening [55]. A recent study used wearable-based actigraphy data from glioma patients and mathematical models of the human circadian clock for quantifying the optimal time of TMZ administration based on external clock time and internal circadian time of the patients [56]. The results indicated that differences in daily rhythms within and between patients caused dosing times to differ more in their internal circadian time than in external clock time.

In a retrospective study, 121 high-grade glioma patients who underwent standard radiotherapy alongside TMZ chemotherapy in the morning or afternoon were selected [57]. The afternoon-radiotherapy group exhibited improved overall and progression-free survival compared to the morning group. In addition, following radiotherapy, the levels of lymphocyte expression in the afternoon-radiation group were higher than in the morning-radiation group [57].

In Neves et al.’s study, *PER2* and *BMAL1* expression were analyzed in U87 glioma cells every 4 h for 24 h [58]. The time point immediately following cell synchronization was considered the first time point (T0). Using two different computational models, it was observed that *BMAL1* and *PER2* have the highest circadian activity at T16 and T8 time points, respectively. Further analysis showed that T7 and T8 are the key time points for transferrin receptor expression. Interestingly, using a confocal microscopy study in U87 glioma cells, the highest intracellular uptake of complexes and p53 protein expression was detected at the T8 time point [58]. Furthermore, assessment of p53 mRNA levels, confirming the transcriptional changes at the protein level, indicated that T16 seems to be an optimum time point for increasing therapeutic efficacy in U87 GBM cells. It was concluded that aligning the treatment with the biological clock of GBM cells may improve GBM therapy approaches [58].

A study reported that U343 glioma cell lines expressing low levels of *PER1* exhibit minor DNA damage when exposed to 12.4 Gy/min X-ray irradiation [59]. Moreover, downregulation of *PER1* is associated with a reduction in apoptosis of the U343 cell population following exposure to X-ray radiation [59]. Administration of radiation leads to a higher death rate in cells expressing high levels of *PER1* compared with other cells [59].

Yassine et al. [60] investigated the effect of the time of administration of radiotherapy on the expression of pro-inflammatory cytokines, clock genes, markers for glial activation, oxidative stress, neuronal activity, and proliferation in the hippocampal neurogenic niche of mice with hepatocellular carcinoma. The results showed that radiotherapy affects the mentioned factors in the hippocampus when administered during the light/inactive phase of mice. The authors concluded that radiotherapy during the active phase of mice may have the highest efficacy on tumor tissue and the fewest side effects on liver tissue and the hippocampus [60].

In contrast, some studies have shown no difference in the therapeutic effects of TMZ, considering various times of administration. In a prospective randomized trial, patients with gliomas were randomized to receive TMZ in the morning before 10 a.m. or in the evening after 8 p.m. [61]. No significant differences in adverse events, quality of life, or overall survival were observed between patients. Geurts et al. [62] evaluated toxicity and survival data in patients with GBM who received TMZ in the morning, afternoon, or evening. No difference was observed in patients’ outcomes. Sapienza et al. [63] also assessed the hypothesis that the time of day in radiotherapy treatment influences outcomes or toxic events in HGG. Yet, they could not see any differences in overall or progression-free survival between patients treated in the morning or the afternoon.

These findings suggest that the benefit of chronotherapy in GBM may be more than selecting an appropriate administration time. The available studies indicate that the response to TMZ may be influenced by the circadian state of GBM cells rather than time of administration alone.

The inconsistent findings likely reflect the complexity of the effect of chronotherapy in GBM. Various parameters, including differences in the chronotype of the patient, tumor characteristics, and treatment schedules, may contribute to the variety in reported outcomes. These observations suggest that the external clock time alone may be insufficient for optimizing treatment scheduling. Moreover, these observations highlight the importance of considering the various characteristics of GBM cells and the internal circadian rhythm of each patient to personalize the treatment and potentially increase the efficacy of chemotherapy and radiotherapy.

Interestingly, an association between circadian rhythms and the integrity of the blood–brain barrier (BBB) has been suggested [64]. Chronotherapeutic approaches considering the timing of drug administration in line with the BBB rhythms can lead to improved drug penetration into the brain and increase the efficacy of treatments of various disorders, including GBM [65]. Furthermore, circadian rhythms affect the pharmacokinetics and pharmacodynamics of chemotherapy and radiotherapy [66]. The distribution of drug transporters across biological membranes is affected by circadian rhythms [67]. The timing of drug administration may influence its absorption, distribution, metabolism, and excretion (ADME) [68]. Clinical findings of chronotherapy in GBM, as indicated in multiple studies [55,61,62,63], appear to be heterogeneous despite their biological rationale.

Overall, the evidence suggests that chronotherapy should not be considered only as administering treatment in the morning or evening. Rather, its effectiveness may depend on aligning the treatment with the internal circadian rhythm of both the patient and the tumor. On the other hand, clock time should not be considered equivalent to biological time. There is variation in individuals’ endogenous circadian phase due to differences in their chronotype, sleep–wake schedules, and light exposure.

Future studies should therefore move beyond simple morning-versus-evening comparisons. Prospective designs incorporating actigraphy [44], sleep-related measures [44], and molecular stratification, including MGMT-related profiles [52], may help identify subgroups with time-dependent differences in treatment response.

Figure 2 shows the schematic representation of the association between chronotherapy and GBM.

## 9. Discussion

It should be noted that some of the cited clinical studies evaluated sleep quality and fatigue in patients rather than directly assessing circadian rhythm or molecular clock function. Nevertheless, these studies can be relevant because SD and fatigue in patients with GBM are common manifestations of CRD [44,69]. In patients with GBM, neurological dysfunctions, medications [12], and radiochemotherapy [70] may contribute to disruption in the sleep–wake cycle. Therefore, although these clinical studies do not directly investigate circadian mechanisms, they indicate indirect evidence of CRD in GBM and other malignancies.

A contradiction exists in the literature regarding the role of *BMAL1* in GBM. Several studies support a tumor-suppressive function of *BMAL1*. It was suggested that disruption in *BMAL1* expression may increase tumor growth [46] and that its deletion abolishes the circadian rhythm in TMZ-induced apoptosis and growth inhibition [51]. In contrast, *BMAL1* was shown to be required for the maintenance of GSCs, and silencing *BMAL1* or *CLOCK* induces cell-cycle arrest and apoptosis [47]. This apparent inconsistency may be due to the context-dependent function of *BMAL1* in GBM. *BMAL1* may exert tumor-suppressive effects in differentiated tumor cells while supporting the survival of GSCs.

In contrast to *BMAL1*, the evidence regarding *PER2* is generally consistent with its tumor-suppressive role in GBM. *PER2* overexpression inhibits proliferation, stemness, and invasion of GSCs and promotes cell-cycle arrest [14]. However, most of the current evidence is derived from in vitro experiments and preclinical studies.

The conflicting findings of different studies may be due to variations in study design and methodology. Differences in factors such as experimental models, patient populations, circadian assessment methods, and treatment protocols may influence the therapeutic outcomes. Moreover, variation in the expression of core clock genes, their different functions, and the molecular characteristics of GBM may contribute to inconsistent results. These factors should be considered when comparing the evidence and may explain the contradiction in the reported beneficial effects of chronotherapy in GBM. In addition, several factors associated with the tumor and the patient, such as MGMT promoter methylation status, chronotype, steroid use, and treatment protocols, may affect therapeutic outcomes.

Future chronotherapy strategies may benefit from using personalized circadian assessment through wearable devices [71]. Since the circadian phase varies among individuals, non-invasive approaches such as actigraphy [56] may help to assess the sleep–wake cycle and circadian components to optimize treatment timing for each patient. Such personalized approaches may be relevant in patients with GBM who experience CRD. Nevertheless, prospective clinical studies are required to determine whether personalized chronotherapy improves treatment efficacy compared with common treatment protocols.

There are caveats to be noted. Although clinical evidence is expanding, a sizeable share of current findings still originates from preclinical studies. In addition, many studies report treatment timing based on clock time rather than biological time, which may contribute to inconsistent findings. Therefore, the current evidence should be interpreted with caution until validated by additional clinical studies.

## 10. Conclusions

The role of circadian rhythms in maintaining physiological processes and molecular mechanisms, including the cell cycle and DNA repair, has been well documented in the literature. The association between CRD and malignancies, including GBM, has also been reported. Alterations in the expression of core circadian genes may contribute to gliomagenesis. Additionally, restoring normal circadian gene function has been shown in preclinical studies to partially suppress GBM progression. In addition, chronotherapy has been proposed as a potentially promising future strategy in GBM treatment. Although it is not reproduced by all studies, aligning chemotherapy and radiotherapy with the internal clock of each patient may represent a potential approach to optimize the efficacy of treatments. Understanding the potential mechanisms underlying the association between circadian rhythm and GBM pathogenesis can facilitate the development of circadian-based therapeutic strategies.

## 11. Future Directions

There is growing evidence regarding the relationship between CRD and GBM. Further experimental and prospective clinical studies are needed to understand the mechanisms underlying how circadian rhythm alterations may lead to tumorigenesis and therapeutic resistance. Assessing CRD in GBM patients using objective circadian measurements rather than relying solely on clock time could be a valuable strategy for personalizing treatment approaches. In addition, the circadian phase of each patient can be assessed using individual parameters such as actigraphy. Furthermore, therapy administration, including chemotherapy and radiotherapy, based on the individual circadian phase of each patient may be beneficial.

## Figures and Tables

**Figure 1:**
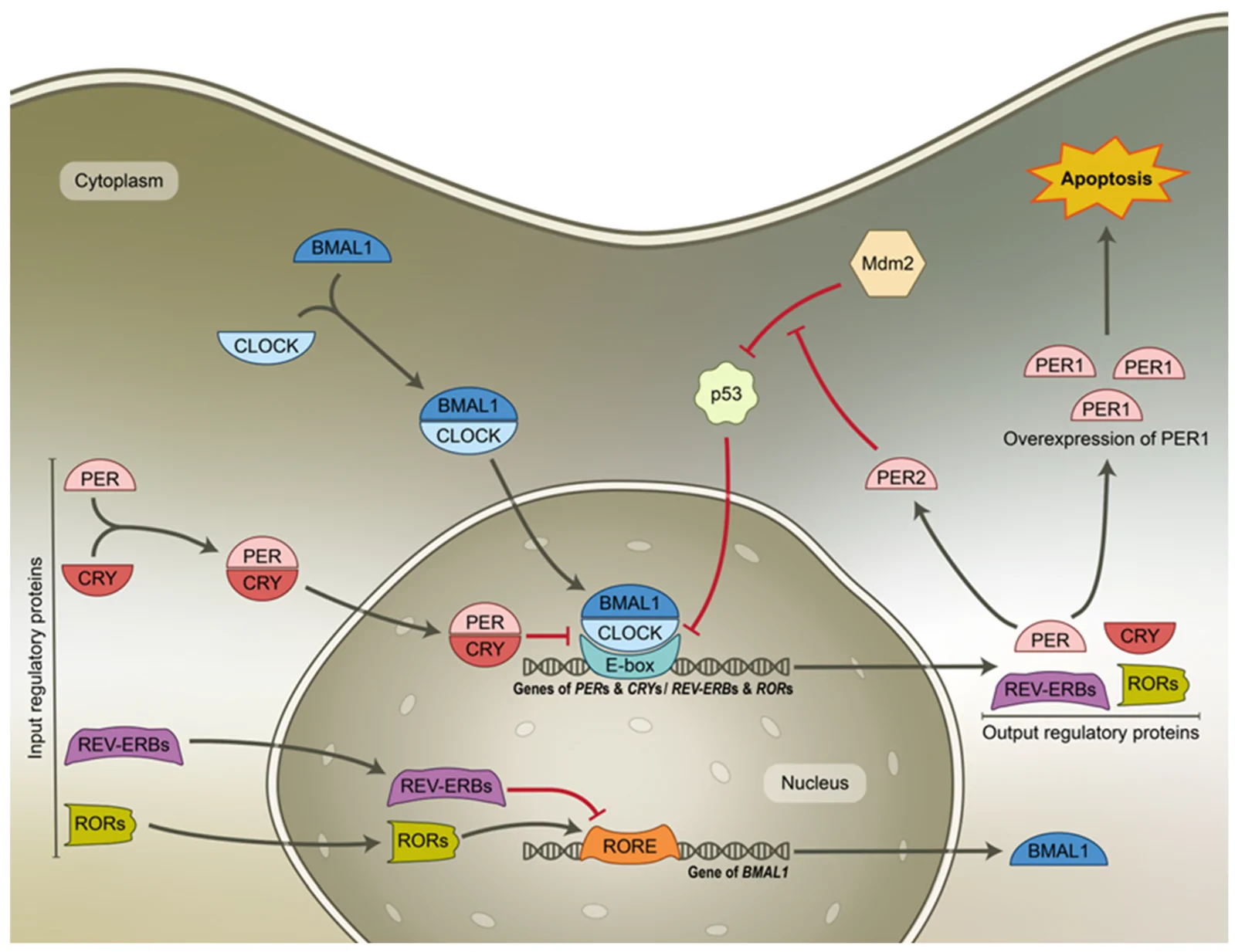
The association between circadian genes and cancer at the molecular level. The molecular basis of circadian rhythms in the SCN of mammals is TTFL, which consists of two activators, CLOCK and BMAL1, and two inhibitors, CRY and PER. The proteins CLOCK and BMAL1 heterodimerize in the cytoplasm and are then transferred to the nucleus. CLOCK/BMAL1 complex interacts with E-box elements on the promoters of target genes, including *CRY*s, *PER*s, *REV-ERB*s, and *ROR*s. PER and CRY proteins form a complex in the cytoplasm, which is then transferred to the nucleus and inhibits the transcriptional activity of the CLOCK/BMAL1 complex. Furthermore, RORs and REV-ERBs promote and inhibit the transcription of the *BMAL1* gene by activating and blocking the RORE binding site of the *BMAL1* promoter, respectively. Additionally, p53 suppresses *Per2* expression by blocking BMAL1/CLOCK binding to the *Per2* promoter. Furthermore, PER2 binds to p53 and prevents Mdm2-mediated p53 degradation. On the other hand, overexpression of PER1 leads to apoptosis in cancer cells. Abbreviations: BMAL1, basic helix-loop-helix ARNT-like 1; CLOCK, circadian locomotor output cycles kaput; CRY, cryptochrome; E-box, enhancer box; Mdm2, murine double minute 2; PER, period circadian regulator; REV-ERBs, nuclear receptor subfamily 1 group D members; RORE, ROR element; RORs, retinoic acid receptor-related orphan receptors; SCN, suprachiasmatic nucleus; TTFL, transcription–translation feedback loop.

**Figure 2:**
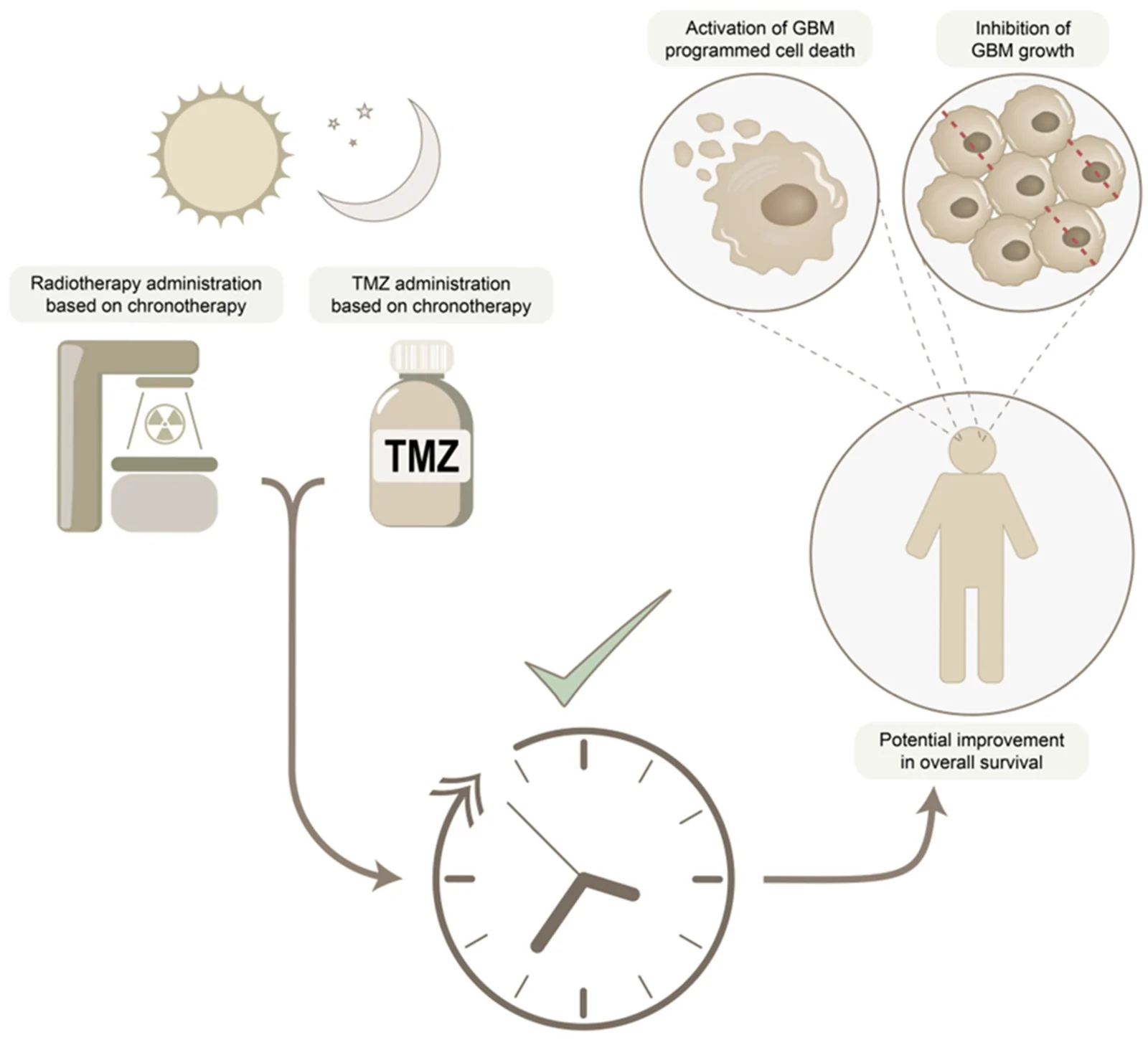
The association between chronotherapy and GBM. The circadian genes contribute to cancer-related biological pathways, including cell growth and programmed cell death (apoptosis). It has been shown that TMZ and radiotherapy administration based on chronotherapy may activate GBM programmed cell death, inhibit GBM growth, and could potentially improve patient overall survival. Abbreviations: GBM, glioblastoma; TMZ, temozolomide.

## Data Availability

No new data were created or analyzed in this study. Data sharing is not applicable to this article.
